# Comparison of Silk Hydrogels Prepared via Different Methods

**DOI:** 10.3390/polym15224419

**Published:** 2023-11-16

**Authors:** Jiahui Hua, Renyan Huang, Ying Huang, Shuqin Yan, Qiang Zhang

**Affiliations:** State Key Laboratory of New Textile Materials and Advanced Processing Technologies, School of Textile Science and Engineering, Wuhan Textile University, Wuhan 430200, China; 13006331906@163.com (J.H.); 15973219031@163.com (R.H.); zhangq12041008@163.com (Q.Z.)

**Keywords:** silk hydrogels, crosslinking, photopolymerization, biocompatibility

## Abstract

Silk fibroin (SF) hydrogels have garnered extensive attention in biomedical materials, owing to their superior biological properties. However, the challenges facing the targeted silk fibroin hydrogels involve chemical agents and shortfalls in performance. In this study, the silk fibroin hydrogels were prepared in different ways: sonication induction, chemical crosslinking, photopolymerization, and enzyme-catalyzed crosslinking. The SF hydrogels derived from photopolymerization exhibited higher compressive properties, with 124 Kpa fracture compressive stress and breaks at about 46% compression. The chemical crosslinking and enzyme-catalyzed silk fibroin hydrogels showed superior toughness, yet sonication-induced hydrogels showed brittle performance resulting from an increase in silk II crystals. The chemical-crosslinked hydrogel demonstrated lower thermostability due to the weaker crosslinking degree. In vitro, all silk fibroin hydrogels supported the growth of human umbilical vein endothelial cells, as the cell viability of hydrogels without chemical agents was relatively higher. This study provides insights into the formation process of silk fibroin hydrogels and optimizes their design strategy for biomedical applications.

## 1. Introduction

In recent years, hydrogels have garnered significant attention in biomedical applications due to their high water-retaining property and their resemblance to extracellular matrix-like three-dimensional networks. Hydrogels can swell rapidly in water and absorb large amounts of water without dissolving, making them more like natural soft tissues than other types of polymeric biomaterials. The three-dimensional network structure of hydrogels can effectively immobilize and release active factors, and their structure and excellent properties are very similar to those of an extracellular matrix (ECM), which provides a favorable microenvironment for cell growth and proliferation [1]. Compared to other materials, hydrogels with designed structures and superior properties offer a favorable microenvironment for cell growth and proliferation. Various hydrogels have been prepared using physical and chemical crosslinking technologies [2]. However, their practical applications are still limited and dependent on the development of appropriate preparation methods. Natural polymer hydrogels hold unique advantages in the field of biomaterials [3,4].

Silk is a natural polymer extracted from silk cocoons. With superior biocompatibility, degradability, and processability, silk fibroin (SF) hydrogel, which is prepared using various processing methods due to SF’s distinctive properties, exhibits great potential in biomedical materials. The traditional process of SF hydrogel formation via bottom-up self-assembly often takes several weeks to months. In order to accelerate the conformation transition of SF macromolecules and achieve fast SF hydrogel formation and water insolubility, techniques such as ultrasonic treatment, pH adjustment, freeze–thawing, and electric fields are employed [5,6]. However, the hydrogels formed by physical crosslinking are achieved via physical interactions such as hydrogen bonding or hydrophobic interactions, which are usually unstable, and the hydrogels formed by chemical crosslinking sometimes have low crosslinking degrees. The resulting hydrogel may suffer from issues of reversible crosslinking and insufficient mechanical properties [7,8]. So, different gelation methods significantly impact the properties of SF hydrogels, including swelling and elastic modulus, among others [9]. Chemical crosslinking, using agents like carbodiimide, Kyanepin, and glutaraldehyde, a phospholipid (1,2-dimyristoyl-sn-glycero-3-phospho-(1′-rac-glycerol) or DMPG), and various alcohols, can create a permanent network structure in SF hydrogels [10,11]. Nonetheless, these crosslinking processes are slow, typically taking more than 4 h, and the crosslinking agents exhibit cytotoxicity and low biocompatibility. In recent years, horseradish peroxidase (HRP) is a commonly used enzyme that produces SF hydrogels with high elasticity, though gelation requires a longer time than the ultrasound SF hydrogel [12]. Hydrogen peroxide often was added to the enzyme crosslinking system to accelerate the crosslinking reaction. Yet, the crystallinity of SF increases with the reaction time, and HRP, as an exogenous enzyme, is cytotoxic in vivo, making it challenging to precisely control the crosslinking degree of the hydrogels [12,13]. Photo-crosslinked SF hydrogels, created by adding a photoinitiator to the SF solution, involve UV-generated free radicals that activate the macromolecular photoactive groups, causing the SF macromolecular chains to aggregate and form an irreversible covalent network structure [14]. This approach allows the quick and easy preparation of SF hydrogels, making it a meaningful technology to build nontoxic and biocompatible hydrogels for biomaterials.

In this study, our aim is to analyze the properties of SF hydrogels and gain insights into the synthesis and design of high-performance silk-based biomaterials. We successfully prepared four different SF hydrogels using sonication induction, carbodiimide crosslinking (EDC), photopolymerization (I2959), and enzyme-catalyzed crosslinking (HRP). The effects of these various physical and chemical crosslinking methods on the structure and properties of SF hydrogels were systematically evaluated.

## 2. Materials and Methods

### 2.1. Preparation of Different SF Hydrogels

*Bombyx mori* raw silk fibers (Huzhou, Zhejiang, China) were degummed in a boiling 0.05% Na_2_CO_3_ solution three times. The degummed silk fibers were then dissolved in a 9.3 M LiBr (60 °C) solution for 2 h (bath ratio of 1:5), and the SF solution was dialyzed in deionized water for 3 days. The resulting SF solution was filtered and centrifuged to remove any insoluble material, yielding a 5.0 wt% regenerated SF solution for further experimentation.

In order to prepare the sonication-induced (US) SF hydrogel, the SF solution (20 mL) was sonicated using an ultrasonic cell crusher (Scientz-II D, Ningbo, China) at an intensity of 200 W for 8 min. For the chemical-crosslinked SF hydrogel, the SF solution was reacted with N-(3-Dimethylaminopropyl)-N′-ethylcarbodiimide (EDC, Sigma, Shanghai, China), 2-morpholinoethanesulfonic acid (MES, Sigma, China), and N-hydroxysuccinimide (NHS, Sigma, China) in an ice bath for 2 h (SF:EDC:MES:NHS = 1.0:0.2:0.1:0.1 (*w*/*w*)), followed by incubation at 4 °C for 24 h. For the photo-crosslinked SF hydrogel, 4 mL of glycidyl methacrylate (GMA, Aladdin, Shanghai, China) was added to the SF solution at 60 °C (100 mL) at a rate of 0.5 mL/min. Stored away from light for 3 h, the resulting SF-methacrylic acid solution was dialyzed at 4 °C for 72 h to remove the remaining GMA and then freeze-dried to obtain methacrylic acid-modified SF (SFMA). A 5.0 wt% SFMA solution with 0.15 wt% photoinitiator 2-Hydroxy-4′-(2-hydroxyethyl)-2-methylpropiophenone (I2959, Aladdin) was injected into a 5 mL syringe and irradiated under a UV lamp (356 nm) for 10 min to prepare the photo-crosslinked SF hydrogel. To prepare the enzyme-catalyzed SF hydrogel, 600 U/mL of horseradish peroxidase (HRP, Aladdin, China), 3.0% hydrogen peroxide (H_2_O_2_, Aladdin, China), and 5.0 wt% of 5 mL SF solution were mixed in proportions of 100:4:1 (*v*/*v*) and reacted for 30 min (at 37 °C) to obtain an enzyme-crosslinked SF hydrogel.

### 2.2. Morphology Observation of the SF Hydrogels

In order to reduce the influence of the freezing process on the internal morphology of the hydrogel, the hydrogel was frozen with liquid nitrogen. In brief, the SF hydrogels without excess water were quickly frozen at −196 °C (liquid nitrogen) for 1 h and immediately freeze-dried for 72 h. The morphology was mapped using a field emission scanning electron microscope (SEM, Hitachi S-4800, Tokyo, Japan) at an accelerating voltage of 5 kV. Before imaging, all samples were affixed to carbon tapes and sputter-coated with platinum.

### 2.3. Structure and Stability Analysis of the SF Hydrogels

In order to investigate the structure and stability of the SF hydrogels. The SF hydrogels were lyophilized and cut into particles (size ≤ 40 μm). Subsequently, they were pressed into plates with KBr for Fourier-transform infrared spectroscopy (FTIR, Bruker, Vertex70, Karlsruhe, Germany) test. The scanning range was from 400 to 4000 cm^−1^. X-ray diffraction of the hydrogels was performed using an X-ray diffractometer (XRD, Philips, X’Pert-ProMPD, Amsterdam, The Netherlands), with Cu Kα radiation (40 kV, 40 mA). XRD patterns were recorded at a 2θ range from 5° to 45°. A thermogravimetric analyzer (TG, STA300 Hitachi, China) was used to examine stability of the SF hydrogels. TG measurements were carried out in a nitrogen atmosphere with a temperature range of 40–800 °C at a heating rate of 10 °C min^−1^.

### 2.4. Physical Properties of the SF Hydrogels

For water content test, we injected the hydrogel into a mold and prepared it into a cylindrical hydrogel with a diameter of 10 mm and a height of 6 mm using UV photopolymerization and other methods of hydrogel preparation described above. The initial weight of the SF hydrogels was recorded as *W*_0_. The SF hydrogels were thoroughly dried at 105 °C, and the resulting mass was recorded as *W*_1_. In order to assess the swelling behavior and water solubility of the SF hydrogels, they were immersed in 0.1 M phosphate-buffered saline (PBS, pH 7.4) on days 1, 2, 3, 4, 5, 6, and 7 in a shaker at 37 °C. When the weight of hydrogels was determined, excess water on their surface was wiped off with filter papers, and the weight was recorded as *W_t_* (in a resting state). The other samples were dried at 105 °C and recorded as *W_i_* (in a shaking state). The water content *X* (%) of the SF hydrogels was calculated using Equation (1), while the swelling rate was determined using Equation (2), and the water solubility was calculated using Equation (3).

*X*(%) = *W*_0_ − *W*_1_/*W*_0_ × 100% (1)

*SR*(%) = *W_t_* − *W*_0_/*W*_0_ × 100% (2)

*WS*(%) = *W_i_* − *W*_1_/*W*_1_ × 100% (3)

For the mechanical property test, the SF hydrogels were prepared in cylinder-shaped samples with a diameter of 12 mm and a height of 10 mm. Uniaxial compression tests were conducted using a texture analyzer (TMS-PRO, West Palm Beach, FL, USA) with a load of 25 N, a deformation rate of 60%, a compression rate of 10 mm/min, and a trigger force of 0.03 N. The SF hydrogels were freeze-dried to become spongy and crushed into flat surfaces, which were placed on a carrier table of a contact angle measuring instrument (OCA15EC, Filderstadt, Germany) and given water. The camera focus was adjusted, and 10 pictures were taken continuously for each sample the moment the water fell. Photos of droplets landing on the sample at the same time were selected.

### 2.5. Cell Culture

In order to compare the cytocompatibility of different SF hydrogels, the wet SF hydrogels, with a diameter of 12 mm, were placed in 24-well plates (Corning Inc., New York, NY, USA) and thoroughly rinsed before sterilization with 75% ethanol for 15 min. After rinsing with PBS, the human umbilical vein endothelial cells (hUVECs) at a density of 2.0 × 10^5^ cells·well^−1^ were seeded into the SF hydrogels, using Dulbecco’s Modified Eagle Medium (DMEM, Gibco, Grand Island, NY, USA) supplemented with 10% fetal bovine serum (FBS, Gibco, USA) and 1% streptomycin–penicillin (Gibco, USA). The cell-seeded SF hydrogels were incubated at 37 °C in a humidified atmosphere and 5% CO_2_ for 2 h to allow adhesion before adding 1 mL of complete medium into each well. Subsequently, the medium was refreshed every 2 days for each sample.

For the Cell Counting Kit-8 (CCK-8) assay, the mixture of CCK-8 and DMEM medium was prepared at a ratio of 1:10 (50 μL CCK-8 and 500 μL DMEM medium). The mixture was incubated on the cell-seeded hydrogels on days 1, 3, and 7. After 3 h of incubation, 100 μL of the reaction solution was transferred to a 96-well culture plate, and optical density (OD) values at 450 nm were measured using a microplate reader (Bio-Tek Synergy HT, Winooski, VT, USA) (*n* = 3). A blank well containing culture medium with the CCK-8 reagent was used as background absorbance. For cell morphology test, the cell-seeded hydrogels were fixed with 4% paraformaldehyde in PBS for 20 min and permeabilized with 0.2% Triton X-100 in PBS for 5 min after culture on days 1, 3, and 7. After blocking with 2% bovine serum albumin (BSA)in PBS for 30 min, all samples were stained with fluorescein isothiocyanate-phalloidin (FITC-phalloidin, Sigma-Aldrich, Chicago, IL, USA) and 4′,6-diamidino-2-phenylindole (DAPI, Sigma-Aldrich, USA). Fluorescent images were acquired using a confocal laser scanning microscope (CLSM; LSM880, Zeiss, Oberhausen, Germany).

### 2.6. Statistical Analysis

All quantitative test samples were analyzed using five replicates. Numerical data are presented as the mean ± standard deviation (SD). In order to determine the statistical significance of the results, one-way analysis of variance (ANOVA) combined with Tukey–Kramer multiple comparisons test was utilized.

## 3. Results and Discussion

### 3.1. Design and Preparation of the SF Hydrogels

**Figure 1:**
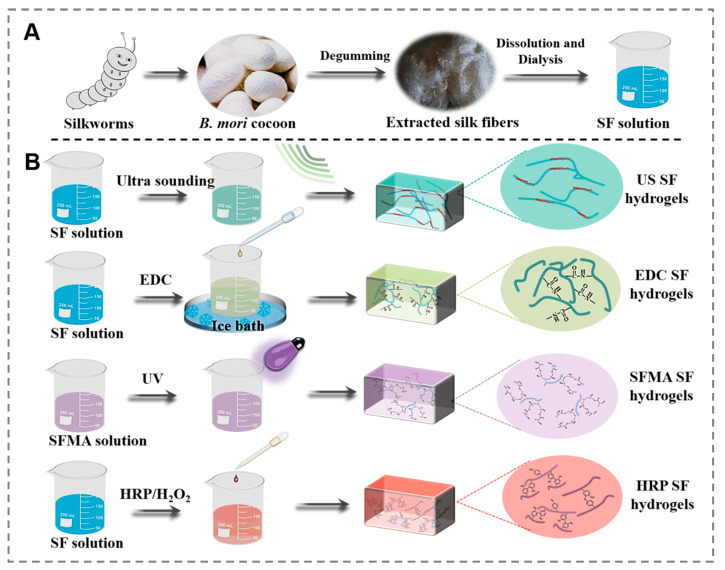
Illustration of fabricated processes of the SF hydrogels. (**A**) Preparation of the regenerated SF solution, (**B**) processes of four types of regenerated SF hydrogels.

In silk-based biomaterials, a well-designed SF hydrogel is key to achieving target performance in its practical application. Although the excellent biocompatibility and processability of regenerated SF material make it a popular precursor for biomedical hydrogel preparation (Figure 1A), some service performance fell short of expectations. The performance of the SF hydrogels is affected significantly by different preparation methods. Herein, four SF hydrogels were prepared in four main ways, resulting in drastically varying gelation states. As shown in Figure 1B, when SF protein was treated with ultrasonic sound, its macromolecular secondary structure would undergo conformational transformation, and a lot of α-helices and random structures transform into β-sheets, which accelerates the sol-gel transformation and promotes the hydrogel formation. A typical chemical-crosslinked SF hydrogel was created by reacting the SF solution with EDC. Depending on the crosslinking of EDC, the amide bond was formed in the form of a covalent bond among SF macromolecules. Once the SF macromolecular network was connected, the SF hydrogels could be formed. In order to accelerate the gelation process, the SF was first modified with glycidyl methacrylate. Through the co-existence of biocompatible Irgacure 2959 [15] and SFMA, the overall crosslinking process was completed with a minimal UV dosage during a significantly reduced crosslinking time. Although ultrasonic and enzyme-catalyzed SF hydrogels are easily handled, the resulting hydrogels still suffer from insufficient mechanical properties and service performance.

### 3.2. Morphology Analysis of the SF Hydrogels

**Figure 2:**
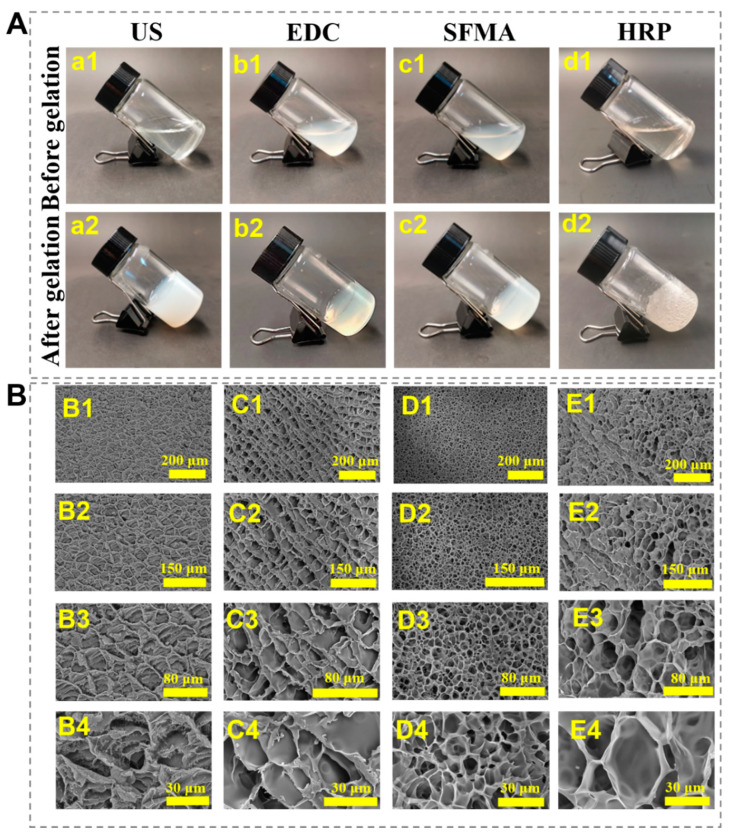
Morphologies of the SF hydrogels formed with different processes. (**A**) Digital pictures of the SF hydrogels: (**a1**,**a2**) for US SF hydrogels, (**b1**,**b2**) for EDC 12 SF hydrogels, (**c1**,**c2**) for SFMA SF hydrogels, (**d1**,**d2**) for HRP SF hydrogels. (**B**) SEM images of the SF hydrogels: (**B1**–**B4**) for US SF hydrogels, (**C1**–**C4**) for EDC SF hydrogels, (**D1**–**D4**) for SFMA SF hydrogels, (**E1**–**E4**) for HRP SF hydrogels.

The time required to form a hydrogel varies between different gelation methods. The US SF hydrogel took 5 min (Figure 2(a1,a2)), the EDC SF hydrogel took 24 h (Figure 2(b1,b2)), the SFMA SF hydrogel took 5 min (Figure 2(c1,c2)), and the HRP SF hydrogel took 10 min (Figure 2(d1,d2)). Both the EDC and the photopolymerized SF hydrogels appeared translucent, while bubbles inside the HRP SF hydrogel, resulting from the production of oxygen, were observed (Figure 2A). In order to reduce the influence of the freezing process on the internal morphology of the hydrogel, the hydrogel was frozen with liquid nitrogen. The freeze-dried hydrogels exhibited interconnected porous structures (Figure 2B), facilitating cell adhesion, proliferation, and the transportation of nutrients and metabolic waste within the hydrogels [16,17]. The pore size of the SF hydrogel was about 20 μm in the ultrasound, chemical crosslinking, and enzymes groups, while it was around 10 μm in the photopolymerization group (Figure S1). This difference is likely caused by the greater degree of crosslinking of the photo-crosslinked SF hydrogels with a denser porous structure. Meanwhile, the different porosity of the hydrogel affects the proliferation and differentiation of cells.

### 3.3. Structures Analysis of the SF Hydrogels

**Figure 3:**
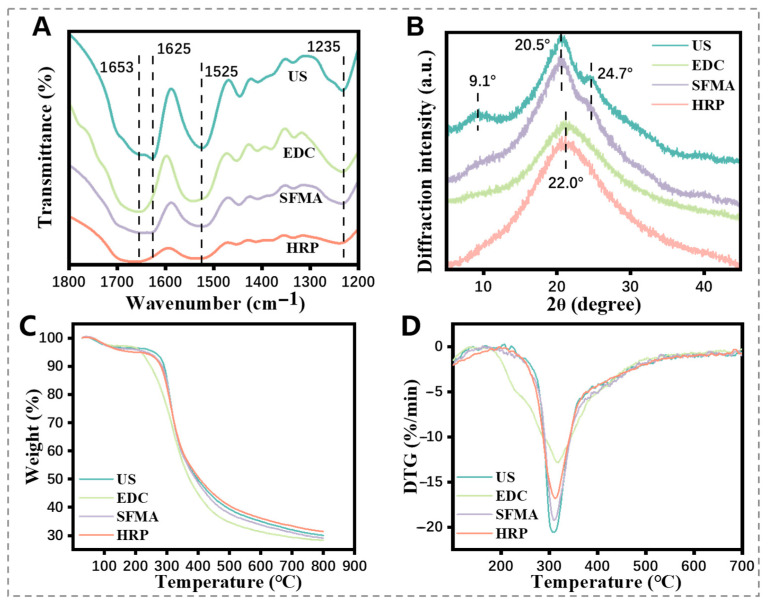
Structural analysis of the SF hydrogels. (**A**) FTIR, (**B**) XRD, (**C**) TG, and (**D**) DTG analysis of different SF hydrogels.

Figure 3A showed that characteristic bands at 1625 cm^−1^ (amide I band) and 1235 cm^−1^ (amide III band) were observed in the FTIR spectra of the US SF hydrogel, which show β-sheet and random structures relatively. Meanwhile, characteristic peaks at 20.5° (silk II) and 9.1° (silk II) were detected via XRD (Figure 3B). These results represented the presence of silk II structures in the US SF hydrogel [18]. The EDC SF hydrogels showed relatively weaker bands and peaks compared to the US SF hydrogel, especially without 1625 cm^−1^ (β-sheet) in the FTIR and the peaks of XRD at 24.7° and 22.0°. The acylated SF hydrogels exhibited prominent peaks at 1235 cm^−1^ and 1525 cm^−1^ in the FTIR spectra, along with strong silk II peaks in the XRD. Previous studies showed that acylation does not affect the secondary structure of the SF hydrogel [19], suggesting that a significant conformational transition occurred in the acylated SF hydrogel after UV polymerization. In the case of the HRP SF hydrogel, the FTIR spectra bands at 1235 cm^−1^ and 1225 cm^−1^ and the XRD peaks at 9.1° and 20.5°, corresponding to silk II structures, were less evident, suggesting that the HRP SF hydrogel had lower crosslinking efficiency during gelation [20,21].

As shown in Figure 3C,D, significant mass was lost before 275 °C in the TG analysis of the US, SFMA, and EDC SF hydrogels, primarily attributed to free water evaporation within the hydrogel. After 275 °C, all hydrogels experienced the degradation of macromolecules, resulting in a remaining mass ranging from 30% to 40%. The EDC SF hydrogel showed a similar profile of thermostability yet exhibited slightly faster degradation and lower remaining mass. It means that in the process of the thermal decomposition of the hydrogel, in addition to the evaporation of its internal water, its structure may have been destroyed at 200–250 °C because of the high temperature of the internal structure, so the weight loss is higher. Consequently, the sonication-induced, enzyme-catalyzed, and SFMA SF hydrogels displayed relatively stable structures, while the EDC SF hydrogel exhibited lower crosslinking degree and structural stability.

### 3.4. Physical Properties of the SF Hydrogels

**Figure 4:**
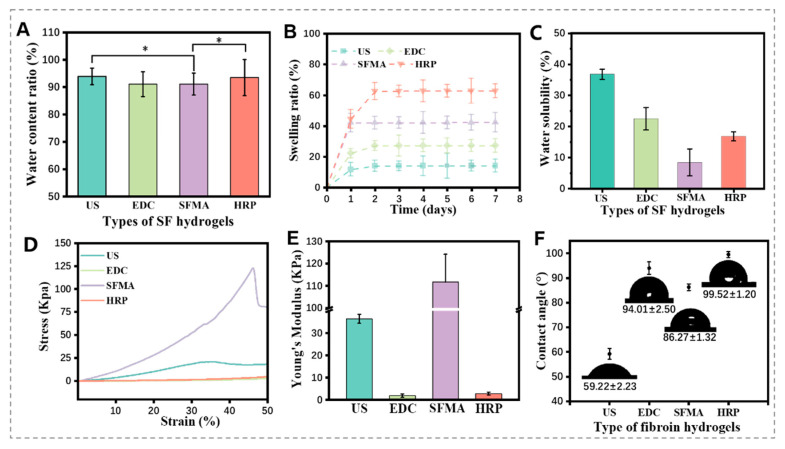
Physical properties of different SF hydrogels: (**A**) water content ratio (* *p* < 0.05), (**B**) swelling ratio, (**C**) water solubility, (**D**) compressive stress, (**E**) Young’s modulus, (**F**) contact angles of different SF hydrogels.

Hydrogel swelling behavior plays a crucial role in its application. Hydrogels with high water content are similar to natural soft tissues and can provide a nutrient-rich environment for cell differentiation and proliferation [22]. Figure 4A showed that the water content of the four SF hydrogels exceeded 90%, indicating high water retention capacity. In the swelling ratio, the US SF hydrogel exhibited the lowest swelling due to its rich hydrophobic β-sheets and larger crystallization zone, without offering more non-crystalline zones and limiting water penetration internally. In contrast, the HRP SF hydrogel showed the highest swelling ratio. The SFMA and EDC SF hydrogels were less affected by the β-sheet structure and exhibited improved swelling performance compared to the US SF hydrogels (Figure 4B). These results highlight the significant impact of crosslinking methods on the swelling behavior of SF hydrogels [23,24,25].

In the hot water dissolution test, the SFMA hydrogels demonstrated the lowest dissolution rate of 8.45 ± 4.33% (Figure 4C), indicating the stable structure of the hydrogel, because it will not disintegrate in a shaking environment. The β-sheet structure is a crucial component governing the mechanical properties of silk-based hydrogels, and its crosslinking degree significantly influences these properties [26,27,28]. High crosslinking enhances strength but reduces the toughness of SF hydrogels by limiting the movement of SF macromolecular chains. The US SF hydrogel showed high thickness but was brittle due to its higher crystallinity, fracturing at around 35% compression (Figure 4D). Conversely, the EDC and HRP crosslinked SF hydrogels exhibited sol–gel behavior with limited crosslinking, and the results in Figure 3A,B show these have predominantly silk Ⅰ structures with low crystallinity, maintaining sufficient toughness and lower brittleness even at 50% compression. The acylated SF hydrogel, with a compressive modulus of approximately 124 KPa, demonstrated the best compressive properties among them. It has higher compressive strength and is fractured at approximately 46% compression (Figure 4E). In the contact angle test (Figure 4F), the SFMA hydrogel exhibited a contact angle of 86.27 ± 1.32°, demonstrating moderate hydrophilicity with relatively suitable water wettability.

### 3.5. Gelation Mechanism Analysis

**Figure 5:**
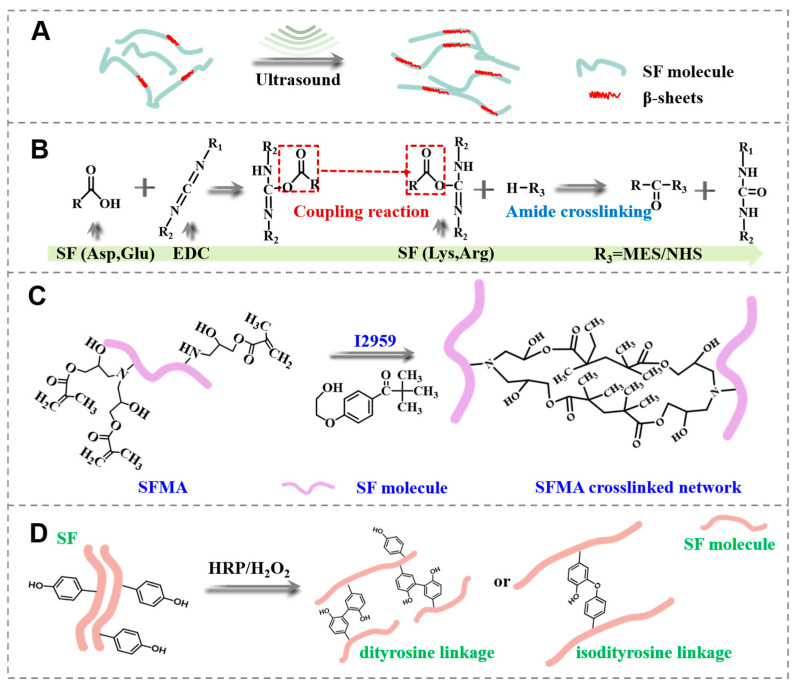
Gelation mechanism of different SF hydrogels. (**A**) US SF hydrogels, (**B**) EDC SF hydrogels, (**C**) SFMA SF hydrogels, (**D**) HRP SF hydrogels.

In this study, four distinct crosslinking processes were employed to prepare SF hydrogels. In the US SF hydrogel (Figure 5A), intense collisions between molecules occur, disrupting the hydrated layer of the SF solution and promoting the aggregation of hydrophobic chain segments under sonication, inducing the formation of β-sheets and gelation [29,30]. The EDC is regarded as a “zero-length” chemical crosslinking agent [31], which reacts with the carboxyl groups of amino acids (e.g., aspartic acid and glutamic acid) on SF, leading to interchain peptide bond formation with adjacent free amino groups (lysine and arginine side chains). The NHS and MES assist the reaction by providing H^+^ under ice-bath conditions during crosslinking, resulting in hydrogel formation (Figure 5B). For the SFMA hydrogel synthesis (Figure 5C), GMA reacts with amino groups on lysine and arginine in SF via an oxidative ring-opening reaction, generating acetyl groups. Additionally, hydroxyl and carboxyl groups in SF macromolecules promote the acylation of serine with GMA via an ester exchange reaction, as a secondary mechanism. The generation of photocurable SFMA forms acylated SF hydrogels under UV light and I2959 excitation [32]. SF contains approximately 10% tyrosine residues, which have phenolic hydroxyl groups that can be oxidized with HRP to form di-tyrosine [33,34,35]. This feature allowed the enzymatic crosslinking by HRP (Figure 5D), combining with H_2_O_2_. HRP catalyzes oxidative coupling reactions, leading to the synthesis of phenolic polymers. Meanwhile, the tyrosine residues crosslink with each other to form the SF hydrogels with di-tyrosine bonds. In summary, the comprehensive properties of different SF hydrogels were considered, and the SFMA SF hydrogel was superior in mechanical properties.

### 3.6. Cytocompatibility of the SF Hydrogels

**Figure 6:**
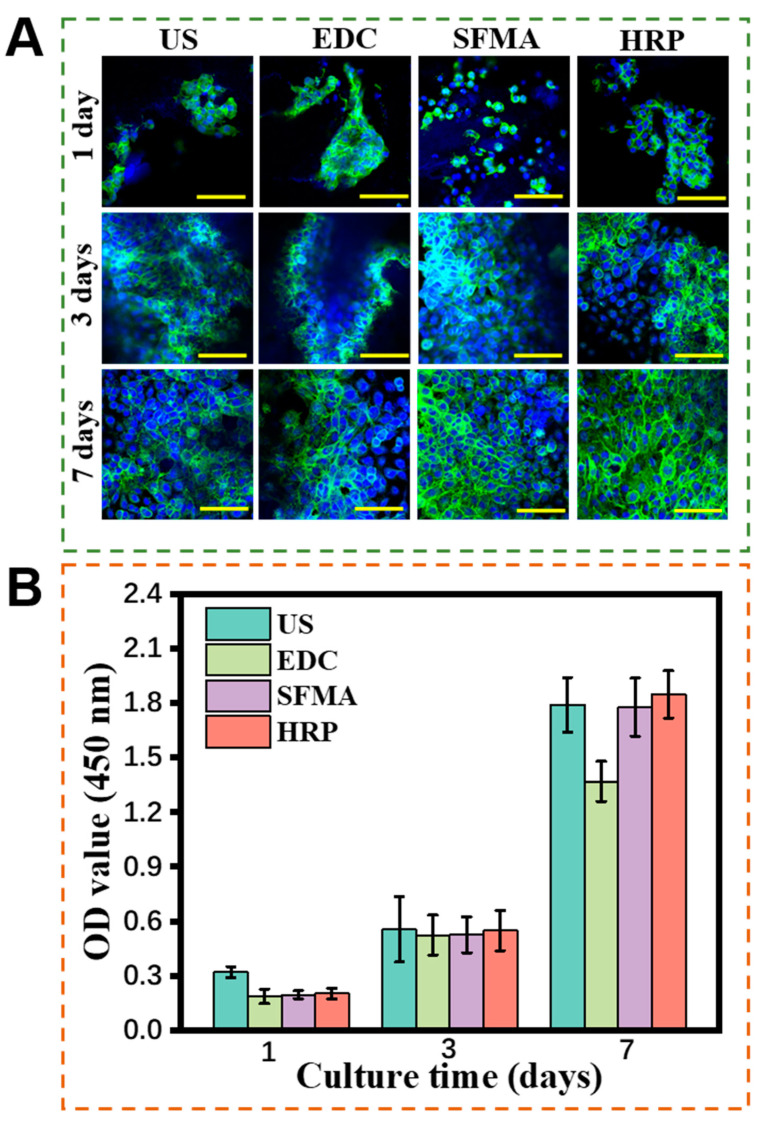
Cytocompatibility of different SF hydrogels. (**A**) Microscopic images of hUVECs cultured onto different hydrogels on days 1, 3, and 7 (scale bar = 100 μm). The cytoskeleton of the cells was stained with green color, and nuclei of the cells with blue color. (**B**) OD values of hUVECs cultured on the SF hydrogels at different time points.

As a biomaterial, cell compatibility is crucial to its biomedical applications [1,2]. The human umbilical vein endothelial cells (hUVECs) were cultured on the SF hydrogels. Figure 6B demonstrated that hUVECs displayed a paving stone-shaped morphology, and their filamentous pseudopods were observed to adhere to the surface of the hydrogels in the four sets of cell-staining images. Cell proliferation continued to increase from day 1 to 7, indicating that hUVECs exhibited high activity on all hydrogels. In contrast, the EDC SF hydrogel showed lower cell proliferation, determined using OD value. Moreover, the acylated SF hydrogels demonstrated excellent cytocompatibility, and the addition of photoinitiators exhibited nontoxic effects on cells.

In order to further visualize cell proliferation on the hydrogels, cytoskeleton, and nucleus staining of hUVECs was performed and observed under a confocal microscope (Figure 6A). Since SF is a natural protein that possesses excellent cytocompatibility, it maintains its superior cytocompatibility after it is prepared in hydrogels. Few cells were present on the first day, sparsely scattered on the hydrogel surface. Notably, the cells proliferated significantly on days 3 and 7, evenly spreading over the hydrogel surface, indicating excellent cytocompatibility for all four SF hydrogels.

In this study, the mechanism of gelation of SF hydrogels involves both physical and chemical crosslinking. Physical crosslinking is achieved via physical interactions. In contrast, chemical crosslinking usually involves reactions between different molecular chains, with the formation of new chemical bonds. These various crosslinking mechanisms make it difficult to standardize the crosslinking degree of different hydrogels. With the complexity and functionalized requirements of hydrogel, SF hydrogels are required to have more excellent properties, such as the preparation of interpenetrating hydrogels, to improve the mechanical properties and structural stability of hydrogels via intermolecular interactions [36,37].

## 4. Conclusions

SF hydrogels have attracted widespread attention in biomaterials. In this study, a comparison of the morphology, structures, and properties of four distinct crosslinking SF hydrogels demonstrates that the sonication-induced SF hydrogel has high compressive properties and stiffness. Moreover, the EDC and HRP crosslinked SF hydrogels displayed high elasticity and porosity. Remarkedly, photo-crosslinked SF hydrogels have superior mechanical properties and excellent cytocompatibility. Their crosslinking degree can be regulated by the photo-polymerization time, which also corresponds to their apparent properties. All hydrogels supported the hUVEC’s proliferation, growth, and utility in biomedical applications. This study provides insights into the preparation of high-performance hydrogels and may offer a useful strategy for bioactive SF hydrogels in biomaterials.

## Data Availability

The data presented in this study are available on request from the corresponding author.

## Supplementary Materials

The following supporting information can be downloaded at: https://www.mdpi.com/article/10.3390/polym15224419/s1, Figure S1: Pore size distribution of four types of SF fibroin hydrogels in dry state. (**A**) Ultrasound, (**B**) EDC, (**C**) SFMA, and (**D**) HRP.
