# Supplementary material for: Comparison of Silk Hydrogels Prepared via Different Methods
Source: Polymers (Basel). 2023 Nov 16;15(22):4419. doi: 10.3390/polym15224419 (PMC10674597; doi:10.3390/polym15224419)
Supplement: Supplementary file 1 [file polymers-15-04419-s001.zip › polymers-2695318-supplementary.pdf]

Supporting Information

Comparison of Silk Hydrogels Prepared via Different Methods

Jiahui Hua, Renyan Huang, Ying Huang, Shuqin Yan*, Qiang Zhang*

State Key Laboratory of New Textile Materials and Advanced Processing Technologies, School of Textile Science and Engineering, Wuhan Textile University, Wuhan 430200, China

Corresponding authors.

E-mails: yingh@wtu.edu.cn (Y. Huang); ysq_zq@163.com (S. Yan)

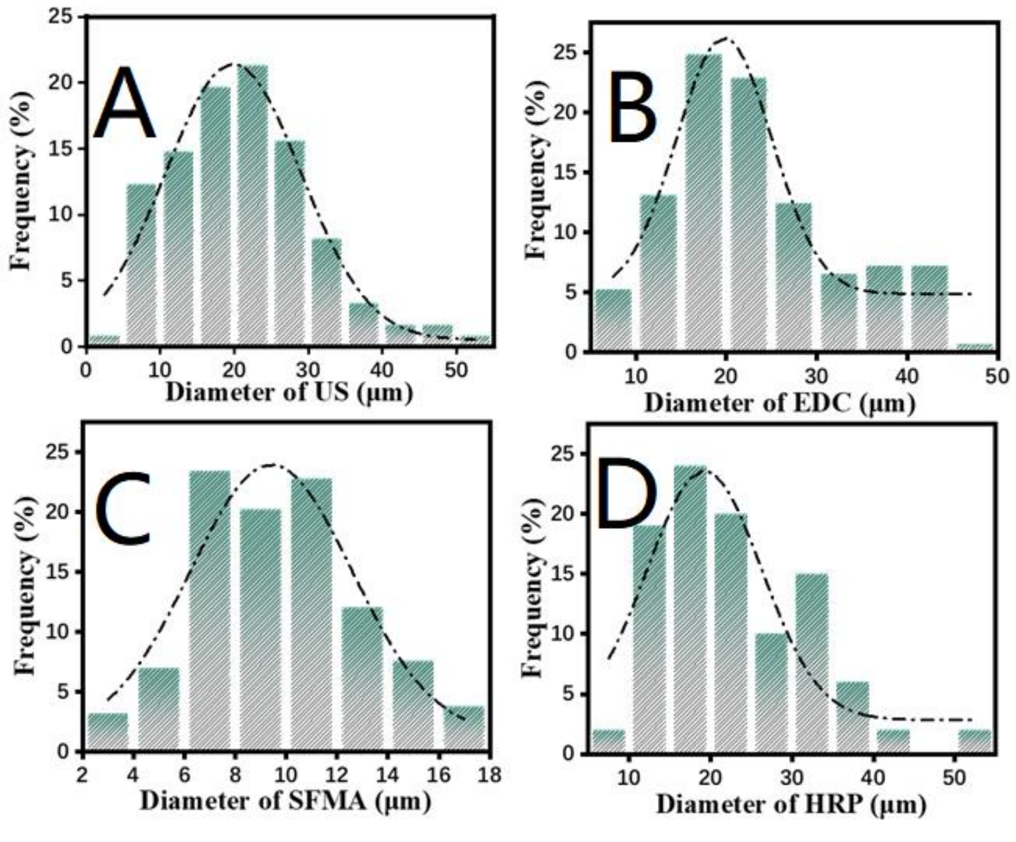

Figure S1. Pore size distribution of four types of SF fibroin hydrogels in dry state. (A) Ultrasound and (B) EDC and (C) SFMA and (D) HRP.
